# Coping Strategies and Social Support in a Mobile Phone Chat App Designed to Support Smoking Cessation: Qualitative Analysis

**DOI:** 10.2196/11071

**Published:** 2018-12-20

**Authors:** Esther Granado-Font, Carme Ferré-Grau, Cristina Rey-Reñones, Mariona Pons-Vigués, Enriqueta Pujol Ribera, Anna Berenguera, Maria Luisa Barrera-Uriarte, Josep Basora, Araceli Valverde-Trillo, Jordi Duch, Gemma Flores-Mateo

**Affiliations:** 1 Centre d'Atenció Primària Horts de Miró (Reus - 4) Gerència d'Àmbit d'Atenció Primària Camp de Tarragona Institut Català de la Salut Tarragona Spain; 2 Facultat d'Infermeria Departament d'Infermeria Universitat Rovira i Virgili Tarragona Spain; 3 Institut Català de la Salut Unitat de Suport a la Recerca Tarragona-Reus Institut Universitari d'Investigació en Atenció Primària Jordi Gol (IDIAP Jordi Gol) Reus Spain; 4 Institut Universitari d'Investigació en Atenció Primària Jordi Gol (IDIAP Jordi Gol) Barcelona Spain; 5 Universitat de Girona Girona Spain; 6 Universitat Autònoma de Barcelona Bellaterra (Cerdanyola del Vallès) Spain; 7 Centre d’Atenció Primària La Granja (Tarragona-2) Gerència d’Àmbit d’Atenció Primària Camp de Tarragona Institut Català de la Salut Torreforta (Tarragona) Spain; 8 Unitat de Suport a la Recerca Tarragona-Reus Institut Universitari en Atenció Primària Jordi Gol (IDIAP Jordi Gol) Reus Spain; 9 Departament de Salut Barcelona Spain; 10 Departament d'Enginyeria Informàtica i Matemàtiques Universitat Rovira i Virgili Tarragona Spain; 11 Unitat d’Anàlisi i Qualitat Xarxa Sanitària i Social Santa Tecla Tarragona Spain

**Keywords:** primary health care, qualitative research, mobile apps, smoking cessation, social support, psychological adaptation

## Abstract

**Background:**

Smoking is one of the most significant factors contributing to low life expectancy, health inequalities, and illness at the worldwide scale. Smoking cessation attempts benefit from social support. Mobile phones have changed the way we communicate through the use of freely available message-oriented apps. Mobile app–based interventions for smoking cessation programs can provide interactive, supportive, and individually tailored interventions.

**Objective:**

This study aimed to identify emotions, coping strategies, beliefs, values, and cognitive evaluations of smokers who are in the process of quitting, and to analyze online social support provided through the analysis of messages posted to a chat function integrated into a mobile app.

**Methods:**

In this descriptive qualitative study, informants were smokers who participated in the chat of Tobbstop. The technique to generate information was documentary through messages collected from September 2014 through June 2016, specifically designed to support a smoking cessation intervention. A thematic content analysis of the messages applied 2 conceptual models: the Lazarus and Folkman model to assess participant’s experiences and perceptions and the Cutrona model to evaluate online social support.

**Results:**

During the study period, 11,788 text messages were posted to the chat by 101 users. The most frequent messages offered information and emotional support, and all the basic emotions were reported in the chat. The 3 most frequent coping strategies identified were physical activity, different types of treatment such as nicotine replacement, and humor. Beliefs about quitting smoking included the inevitability of weight gain and the notion that not using any type of medications is better for smoking cessation. Health and family were the values more frequently described, followed by freedom. A smoke-free environment was perceived as important to successful smoking cessation. The social support group that was developed with the app offered mainly emotional and informational support.

**Conclusions:**

Our analysis suggests that a chat integrated into a mobile app focused on supporting smoking cessation provides a useful tool for smokers who are in the process of quitting, by offering social support and a space to share concerns, information, or strategies.

## Introduction

### Background

Smoking is one of the most significant factors contributing to low life expectancy, health inequalities, and illness at the worldwide scale [1]. Every year, tobacco kills approximately 6 million people and causes economic losses in the order of half a billion dollars. However, the deaths caused by smoking are the most preventable and, as a World Health Organization report points out, the impact of the tobacco epidemic can be reduced by using low-cost, high-efficiency means [1]. According to data from the 2014 European Health Survey, conducted in Spain by the National Institute of Statistics, 30.4% of men and 20.5 % of women are smokers, compared with the European mean of 21.9% and 15.1%, respectively [2].

### Online Support for Smoking Cessation

The rise of internet use and mobile phones has introduced 2 key features in the way we communicate with each other: communications are now (1) ubiquitous, that is, you can talk with almost anyone anytime (24 hours a day) anywhere and (2) nearly instantaneous, as messages can be received and answered within seconds or minutes. Moreover, individuals can be connected at minimal cost, eliminating barriers to in-person participation in group programs such as childcare, disability, and employment responsibilities [3].

The current guidelines recommend that all smoking cessation programs incorporate some type of social support [4]. This may include social networks and mobile communication–based systems that provide a platform where those trying to quit smoking can share concerns and offer emotional support, useful advice, personal stories, and reinforcement during all the smoking cessation process [5]. Online support groups also offer a degree of anonymity that would not be possible in face-to-face communication, which may encourage individuals to openly discuss their experiences without fear of a negative reaction [6].

However, little is known about the efficiency and the importance of online support in smoking cessation programs. To our knowledge, previous studies published about this topic found that the support of social networks may be beneficial immediately when smokers want to quit and also during the first weeks of a smoking cessation program [3,7].

### Conceptual Models

Lazarus and Folkman model defines the concept of stress by referring to the interrelationships that occur between a person and the context in which that individual finds himself or herself. The Lazarus and Folkman model may be transferred to the smoking cessation field to study psychological factors. Their model suggests that anxiety levels depend on the ability to handle external demands and internal evaluations that exceed the resources of the individual and on the strategies used to cope with them. This framework is appropriate for this study because smoking cessation is considered an important stressful factor. Although most smokers aged 18 years or older expressed a desire to quit and 52% had attempted to do so, only 6% of them had successfully quit at 12 months. Previous studies have found stress-induced craving response to be particularly important in smokers with high levels of nicotine dependence, who may be at greatest risk for cessation failure [8,9]. Stress-coping progams increase success in quitting smoking [8,9].

The Lazarus and Folkman model addresses 6 categories [10]: (1) emotions, for which our study applied the Ekman classification of primary emotions (joy, sadness, anger, disgust, fear, and surprise) [8]; (2) coping strategies, both task-oriented and emotion-oriented; (3) beliefs, defined as preexisting notions of reality, whether individually created or culturally shared, and in this case, referring to the smoking cessation process; (4) values, encompassing the objectives that express what is important to the individual and will help him or her to quit smoking; (5) cognitive evaluation, a process that determines the consequences a particular event will generate in an individual; and (6) social support, a coping resource whereby someone provides emotional, informative, and/or tangible support.

The discussion of emotions is a key element of online support groups. Cutrona and Suhr developed a coding scheme to classify social support behaviors as emotional, informational, self-esteem, social network, and tangible support [9]. They identified all 5 types of social support in online posts, with informational and emotional support most frequently observed. People who decide to quit smoking may benefit from having developed coping strategies to overcome the habit.

### Tobbstop Trial

The Tobbstop trial was a multicenter randomized clinical trial (Registration: clinicaltrial.gov NCT01734421) carried out in Tarragona, Reus, and surrounding areas in Catalonia (Spain) that aimed to assess the efficacy of a mobile phone app for smoking cessation. Smokers were recruited from primary health care centers and were randomized into 2 groups: (1) an intervention group that included access to the Tobbstop mobile app and the usual counseling about smoking cessation provided in primary health care consultations [11] and (2) a control group that received only the usual smoking cessation counseling.

This study analyzed one of the components of the Tobbstop app, a private chat that allowed study participants to communicate with each other [12]. The objectives of this study were to identify emotions, coping strategies, beliefs, and values, together with cognitive evaluation of smokers during the process of quitting, and to analyze online social support provided through messages posted to this chat.

## Methods

### Design

Descriptive qualitative study to identify the emotions, motivations, and perceived benefits that could be observed in daily experiences within the process of change experienced by people who used this chat function during the action phase of the change process.

### Participants

Of the 309 participants randomly selected for the intervention group, 102 participated writing comments in the chat, constituting our study population. The sample was opportunistic [13]. Inclusion criteria were being adults (older than 18 years) with a motivation ≥6 points on the Richmond test [11], in the action phase according to Prochaska and DiClemente model of change [14], and who had an iOS or Android-based mobile phone.

The Prochaska and DiClemente model describes stages related to addictive behaviors in individuals trying to abandon substance use. The stages according to this model are precontemplation (denial a problem exists), contemplation (self-awareness of problem begins), preparation stage (individual starts making concrete plans to abandon substance use), action stage (reduction and cessation of smoking), and finally, a maintenance stage.

### Description of the Mobile App

The Tobbstop app was designed to support participants during the first 3 months of the smoking cessation progress, with 3 main goals in mind: (1) to help individuals record their progress in the smoking cessation program; (2) to increase the user’s knowledge about the problems related to smoking and the health benefits associated with smoking cessation; and (3) to provide distraction for moments of craving.

The Tobbstop app included 4 components: (1) a library with information about tobacco; (2) a private chat for study participants where they could ask for help, share concerns, or offer help to others; (3) a set of minigames designed specifically to entertain and educate participants; and (4) a progress registry to show the evolution of the participant’s health throughout the treatment process. The app also included a panic button and consultation with an expert.

### Technique to Generate Data

The technique to generate information was documentary through written text messages. During the Tobbstop study period (September 2014 to June 2016), 11,788 text messages were written in Catalan and Spanish by participants. These were downloaded into an Excel table for analysis, replacing personal information about the participants with identification codes that protected anonymity.

### Analysis

A thematic content analysis of the messages posted in the chat was performed by 2 members of the research team (EGF and GFM) as follows: (1) an initial reading of all messages; (2) identification of relevant topics and text messages; (3) fragmentation of the texts into units of meaning; (4) codification of texts by topics; (5) creation of categories based on the Lazarus and Folkman and the Cutrona model, grouping the codes; and (6) interpretation of the meanings of each category. Analysis was conducted with the support of the ATLAS.ti 7 program.

### Criteria of Rigor and Quality

To ensure the rigor and quality of the study, the following criteria of rigor suggested by Calderón were followed: epistemological and methodological adequacy, relevance, validity, and reflexivity [15]. The context, the characteristics of the participants, and the research process were described. The messages obtained were analyzed, and a period of reflection was carried out by 2 members of the research team.

### Ethical Aspects

The study entitled “Efficacy of an application for mobile devices in smoking cessation in young people (Smart_Smoke): a cluster-randomized clinical trial” was approved by the ethics committee of Instituto de Investigación en Atención Primaria (IDIAP) Jordi Gol (P12 / 041). The app used was called Tobbstop.

Participants voluntarily agreed to participate and provided their signed informed consent. The research team coded the stored messages with an identification number to guarantee confidentiality and protection of the participants’ identity. No names were used in the reported quotations.

## Results

**Figure 1:**
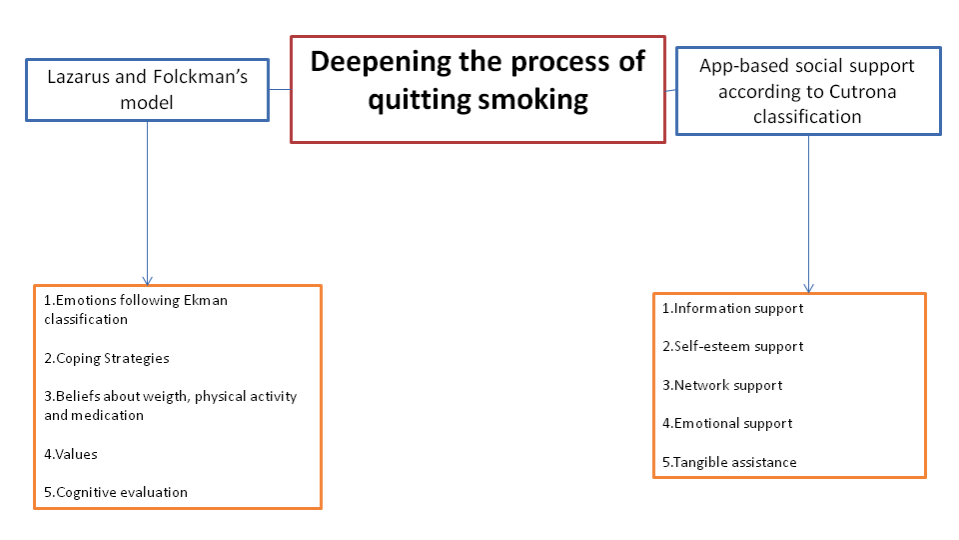
Deepening the process of quitting smoking.

Table 1 shows the characteristics of the participants included. The results are structured in 2 blocks according to the Lazarus and Folkaman model, and Cutrona model categories (Figure 1).

### Lazarus and Folkman Model

On the basis of Lazarus and Folkman model, analysis revealed the following 5 main categories:

**Table 1:** Sociodemographic characteristics of users of the Tobbstop chat (N=102).

Chat participants		Statistics
**Sex, n (%)**		
	Male	59 (57.8)
Age (years), mean (SD)		45.3 (8.9)
**Civil status, n (%)**		
	Single	21 (20.8)
	Married	61 (59.2)
	Widower	2 (2.5)
	Divorced	18 (17.5)
**Educational level, n (%)**		
	No schooling	0 (0.0)
	Primary	26 (25.7)
	Secondary	57 (56.4)
	University or higher	17 (16.8)
Age started smoking (years), mean (SD)		16.1 (3.1)
Number of quit attempts**,** median (IQR^a^)		2 (1-3)
Maximum months of smoking abstinence, median (IQR)		2 (0.7-10.5)

^a^IQR: interquartile range.

#### Emotions

Participants express positive and negative emotions related to smoking cessation process following Ekman classification [8]: fear, surprise, disgust, sadness, joy, and anger. At the beginning, they send messages of sadness and as the time passes, the messages are more positive. The emotion that appeared most frequently at the beginning was fear such as a fear of facing certain social events at which they usually would have smoked. Once these events have passed without falling back into the habit, they showed the joy they felt at remaining abstinent:

I have a dinner tonight and I’m really scared about it.

ID 548, woman, 56 years

Well, I passed an important test, a calçotada party with friends.

ID 399, woman, 58 years

The emotion of surprise also appeared when a participant realized he or she had not thought about tobacco for a long time:

...which means that I did not have time to think about smoking, and even I was surprised.

ID 422, woman, 57 years

I think I’m the newbie here, but it is truly surprising, the changes you notice, from smell to taste...and I’m only on my fifth day!

ID 192, man, 41 years

Some people resort to the emotion of disgust to avoid smoking again:

I pressed the panic button because I would like one cigarette. I’m not so well today.

ID 452, woman, 54 years

As the following dialog shows, in the early stages of the cessation process, sadness emerged as a powerful emotion and there was a sort of a duel about quitting smoking, a habit that had been with them for a long time. This mourning for what participants got out of smoking is also observed in these statements acknowledging that the pleasure of smoking was because of addiction:

I wonder if other people have also felt sad, thinking how happy they were to smoke and now not smoking...I enjoyed the ‘misbehavior’ of smoking...

ID 647, woman, 41 years

Yes...I didn’t even want to get out of bed because I thought, What will I do if I don’t smoke???

ID 548, woman, 54 years

And did you get over it?

ID 647, woman, 41 years

Oh, sure. But it will take you a few more days yet.

ID 548, woman, 54 years

Once the first few weeks have passed, participants reported an immense joy. Many were counting the days that they had gone without smoking and expressed pride in their achievement:

Happy Wednesday. Today makes 120 days I have not smoked. I am very happy and proud of what I’m doing. Don’t give up, everybody!!!

ID 548, woman, 54 years

The Tobbstop app asks about emotions (anger, sadness, and bad or happy mood) every day when individuals start the app. Emotions are dynamic and responsive, and some emotions can be replaced by others throughout the smoking cessation process. The negative emotions appeared in the first days when participants started to quit smoking:

Every time I open the application and it asks me how am I...the first days I answered I am angry, sad, badmood...and now I have been saying for some time that I am happy.

ID 543, woman, 54 years

#### Coping Strategies

External and internal demands assessed as excessive or overwhelming might be confronted with different coping strategies. The most frequent coping strategy identified to decrease psychological stress, anxiety, and fear of relapse was physical activity. Other strategies used to decrease psychological distress and avoid thinking about tobacco consumption included listening to music, cleaning, reading, cooking, or playing:

Stationary bicycle...to not think about tobacco.

ID 279, woman, 40 years

All I do is clean and listen to music to not think about it.

ID 429, woman, 40 years

I fix supper and spend time on that.

ID 406, man, 52 years

When I get overwhelmed I look for a game and I get over it.

ID 363, woman, 54 years

Eating certain foods, drinking liquids, and eating candies as a distraction were also used as a way to reduce stress:

I’m sure that sunflower seed salesmen are happy about my not smoking!!!! I’ve already eaten 2 packages today!

ID 192, man, 41 years

I don’t know how to get over that need, I’m trying right now to think about other things and I am at work but I would really like to smoke. I am going to drink water or a Coca-Cola I might find around the office.

ID 259, woman, 42 years

In order to decrease symptoms of nicotine abstinence, the participants used different types of treatments such as Varenicline, a nicotine substitute. The participants explained the difficulties they experienced with nicotine substitutes to calm the anxiety produced by not having nicotine, especially patches in the case of those receiving no treatment or a different treatment:

I take the pills but now the doctor called to give me the patches because I had a problem with Champix but the truth is that they work.

ID 399, woman, 58 years

For now, the patch gives me the nicotine I need...I only miss having a cigarette between my fingers...

ID 266, woman, 51 years

The following dialog shows that humor is another strategy that was frequently used to reduce stress and anxiety:

So, how’s the car repair going?

ID 647, woman, 41 years

No defects. Hahaha.

ID 485, man, 49 years

And the O2 buffer?

ID 647, woman, 41 years

Bad joke, no? “carboximetry at 2.”

ID 485, man, 49 years

Tell the nurse to send the carbon dioxide meter out for repairs. It must not be working right...

ID 422, woman, 57 years

At follow-up visits, participants tested their carboximetric level; seeing a score of 0 became an element of self-reinforcement. In addition, they thought that a low (or 0) score meant they had clear lungs:

Today at the exhalation test I almost jumped out of the chair, I was so happy. I never thought I’d react like that!

ID 422, woman, 57 years

I went to see the nurse and I got a “2” on lung toxicity.

ID 491, woman, 44 years

#### Beliefs About Weight, Physical Activity, and Medication

Beliefs are cognitive configurations individually created or culturally shared preexisting concepts of reality that act as a perceptual lens. Despite being counseled to follow a healthy diet and drink a lot of liquids, the chat participants showed a belief that it would be impossible to avoid gaining weight during the smoking cessation process. If the timing coincided with menopause and other aspects of aging, they believed there would be greater weight gain:

Did you gain weight too?

ID 694, woman, 50 years

Five kilos (10 pounds) in three months!! But it was worth it. Later on you can lose them, but slowly.

ID 577, woman, 43 years

Age is helping me [gain weight] too...

ID 548, woman, 54 years

That could be.

ID 577, woman, 43 years

Well, with menopause besides, I can’t tell you what all else is going on with me, hahaha.

ID 422, woman, 57 years

Nonetheless, they believed it was worse to smoke than to gain weight and that they would not be able to lose the weight while trying to quit smoking. Instead, they proposed it as a challenge for the near future:

The weight doesn’t worry me too much if we don’t put on too much! Anyway, better 10 extra pounds than smoking again, no?

ID 192, man, 41 years

Yeah, we’ll get rid of the kilos and we will also be rid of the addiction to tobacco.

ID 399, woman, 58 years

Participants believe that physical activity is positive and useful to decrease craving symptoms. Most participants explained that they were exercising regularly:

The truth is that doing sports helps a lot to overcome this vice.

ID 192, man, 41 years

Doing sports is the best !!

ID 164, woman, 32 years

Although many participants were quitting smoking with the help of medication, there was still a belief that it was best to do it without any pharmacological help:

Anyway, if you can leave without anything, that’s the best. It's just a head issue. Be strong. And say no. In a week, the cold turkey effect just no longer exists.

ID 164, woman, 32 years

I don’t know anyone who has used medication to quit smoking. My friends have quit with nothing.

ID 429, woman, 40 years

Participants believe that being in a tobacco-free environment would help them in their quit process and were concerned when they faced situations where they normally would have smoked and where they knew they would meet other smokers:

I’m lucky that my circle of friends does not smoke, almost nobody.

ID 499, woman, 47 years

My son and daughter-in-law also quit a few years ago. My daughter sees it as more difficult for her partner, who also smokes.

ID 422, woman, 57 years

#### Values

Values are expressions of what is important for that person. The most important value expressed by participants was *health*, which was stated as the main motivation to quit smoking:

I want to quit for my health and for my wife and my son. If I get sick they will have a very hard time.

ID 406, man, 52 years

I’m quitting because I do not want the doctor to tell me one day, either quit or you will die.

ID 470, woman, 57 years

Not even 24h yet but I am happy because I need to quit smoking. To health!!!

ID 548, woman, 54 years

They also highlighted the benefits of smoking cessation for their health. They described benefits they perceived in their body, how food smelled and tasted better, how they did not run out of breath while exercising, and how the various follow-up tests reinforced their decision to stop smoking:

but it really is surprising, the changes you notice, from the sense of smell to taste...and I’m only on my fifth day!

ID 192, man, 41 years

I can breathe better, I can smell better AND I smell better. Food tastes better, and I’ve saved 280 euros.

ID 164, woman, 32 years

*Family* was another important value and one of the main motivators to start the process of smoking cessation. Concerns included having a negative influence on their children, grandchildren, or other relatives, or impairing the health of family members, especially children, with second-hand tobacco smoke:

My 4-year-old granddaughter, whose parents do not smoke, saw me and her aunt smoking, and told my daughter-in-law that “when I grow up I’m going to smoke like yaya and auntie.” You should have seen my face, and I told her that I would not smoke anymore because it makes ‘owies’.

ID 422, woman, 57 years

I have 2 little children who have a lot of bronchitis and the smoke is really bad for them...and then for my health...I’m 33 years old and I have a lot of breathing problems.

ID 843, man, 35 years

In other cases, the family had asked them to stop smoking, but the participants were not always receptive to these messages when they were in the early precontemplative stage. They still did not see that smoking would cause any harm and it was not until the contemplative and preparatory stage when they became aware of all the messages they had gotten from relatives, health professionals, and friends:

My two children asked me to quit and I am doing it for them.

ID 280, man, 55 years

My husband is anti-smoking and has been telling me to quit for 9 years and I paid no attention; then one day, looking at Facebook, I saw [the TOBBSTOP study] and I decided.

ID 429, woman, 40 years

Another important value was *freedom* when participants tired of being dependent on tobacco and became conscious of their nicotine addiction. Once they had started the cessation process and were aware they had regained their freedom, this awareness became an important motivator to succeed. They were also critical of people who continued to smoke:

First of all, I didn’t smoke because I liked it but because I was addicted. Tobacco tastes bad.

ID 485, man, 49 years

I’m tired of being dependent on tobacco.

ID 577, woman, 30 years

I won’t go back to it because even when I smoked the monkey stayed on my back. Many times, right after I smoked, I thought, “Really? Again? and I would have smoked another one...”

ID 647, woman, 41 years

Look, yesterday I was inLes Gavarres and for the first time I was inside, not out on the terrace, and I looked at the smokers who were out there. The image was grotesque to look at. It was like they were being controlled. Think about that.

ID 337, man, 41 years

Another value was to *help others to quit smoking*, especially friends and family:

I have convinced three people in my circle to quit...I’m on a crusade against tobaccoooo!

ID 192, man, 41 years

*Money* was a great motivator to begin a cessation attempt although it was combined with other elements. Saving money was an important value in remaining abstinent. The participants talked about what they wanted to buy with the money saved:

I don’t want to smoke -- for my health, the money, the smell on my clothing...

ID 541, woman, 56 years

With what we spent on tobacco, my partner and I could take a cruise you won’t believe...

ID 364, woman, 49 years

#### Cognitive Evaluation

Cognitive evaluation is the process that determines the consequences of smoking cessation in the individual. When participants smoked a cigarette or just took a drag, they were less active in the chat group because they felt sad, guilty, and ashamed although the group encouraged them to continue trying:

I keep reading you...but I have not been able to quit.

ID 259, woman, 42 years

I feel bad because I wear the patch and don’t smoke cigarettes (it bothers me) but I use the electronic cigarette. With non-nicotine liquid and I don’t inhale. I don’t inhale the smoke but I use it and feel guilty about it.

ID 399, woman, 58 years

Although they were aware that the first days are the most difficult, and are when it is easiest to have a relapse, in some cases, they minimized the risk they had overcome during the first weeks:

No prob, man. Once you get through the first week and say a few times, “No, I don’t smoke anymore,” that’s it.

ID 192, man, 41 years

So, yeah, it’s true that it’s hard at first but after that it’s not.

ID 422, woman, 57 years

### Cutrona Model

According to the Cutrona model, online social support was classified into 5 subcategories:

#### Information Support

Chat was perceived by participants as a strength, as it provides cognitive support by sharing advice and practical information with others. Many of the messages offered suggestions about not gaining weight during the cessation attempt:

My advice is to be careful; enjoy the food, which will taste better than ever...but do not forget that you can go from gaining 4 kilos to 10 without even noticing.

ID 164, woman, 32 years

Try with natural juices and sport. Cheer up!! These are the first few days.

ID 429, woman, 40 years

In many messages, participants recommended physical exercise as a method to control anxiety and described the different activities they performed:

The trick is to make up your mind that you really want to stop and do some sport. Try a “fun run” event and I’m sure you will get hooked on it.

ID 192, man, 41 years

Sport or physical activities works well !!!

ID 162, woman, 32 years

Among the advice given to help overcome the withdrawal syndrome was natural remedies (eg, herbal teas and tryptophan):

It is important to stand firm and not smoke or take even one puff. Lime-blossom or valerian tea can help you.

ID 623, woman, 37 years

Often the participants had made previous attempts to quit smoking, and they shared these experiences with the group, including the reasons that led them to relapse. They warned the others not to smoke even a single cigarette because that was what led them to fail:

From all this I learned that if you stop smoking you should never smoke even one.

ID 363, woman, 54 years

You’re right. I did not smoke with the pregnancies and then people offered me one and I went back to smoking.

ID 422, woman, 57 years

Some messages referred to the opinions and advice of experts, sometimes with verbatim phrases of what a doctor had told them:

As my doctor says, quitting smoking is learning and there is no learning without relapses.

ID 320, woman, 50 years

#### Self-Esteem Support

The group offered compliments to those who were achieving their goals and considered them to be role models:

Thank you, you have become champions!

ID 422, woman, 57 years

Congratulations!!! A good example to follow.

ID 477, woman, 30 years

#### Network Support

Recurrent messages were found to provide group support to overcome the worst moments, especially in the first few days. In the group, people found others who were in the same situation and understood what was happening to them at that time:

It’s my second day. I’m having a nervous breakdown!!!!

ID 354, woman, 43 years

Hang in there! It’s my FIRST day and I don’t have to tell you anything you don’t already know.

ID 348, woman, 42 years

Together we’ll make it through!

ID 363, woman, 54 years

The chat also allowed those who were just beginning the program to ask questions to, instead of those who had been in the process longer:

[NAME], I have a question. After 103 days, do you still think about smoking?

ID 355, man, 42 years

#### Emotional Support

Participants sought support from the group when they felt a need to smoke, and received messages of support and motivation to help them get past the *craving* episodes [16]:

I need a cigarrooooo.

ID 375, man, 36 years

Don’t smoke, you are stronger than that.

ID 361, woman, 34 years

They often needed to validate their emotions with the group, especially when they had not smoked for a number of days. It became important to count the days without smoking and to seek congratulations from the group; this positive feedback rewarded them emotionally:

83 days without smoke.

ID 485, man, 49 years

Congratulations!

ID 548, woman, 54 years

The group also offered encouragement when participants relapsed and had a cigarette:

Don’t worry, try again.

ID 361, woman, 43 years

Don’t believe that more than one hasn’t had a fall and still do; they are not all so strong.

ID 422, woman, 57 years

Several participants mentioned eating snacks to quell anxiety. In these cases, the group downplayed the weight gain, considering smoking to be worse than gaining a few extra pounds:

Relax, the extra pounds go away but your lungs and your body in general will thank you...

ID 270, man, 35 years

Several messages show virtual affection:

You’re welcome. When you get the urge to smoke, think “maybe later” and that’s how you get past it. [Sending you] a kiss.

ID 422, woman, 57 years

I am so sorry...there are situations that require your energy...When you start again, you will achieve it, and will do better with experience! Asuper-hug!

ID 477, woman, 30 years

Thank you. Everybody in this group is super-cool!!!

ID 548, woman, 54 years

#### Tangible Assistance

First of all, the group decided to make closer contact and a *whatsapp* group was proposed:

We could do a whatsapp group.

ID 299, woman, 49 years

Yes, that would be cool.

ID 192, man, 41 years

Great, so who’s going to do it?

ID 299, woman, 49 years

If you want, I’ll set it up.

ID 192, man, 41 years

As new participants were being integrated into the chat, they were invited to join the group:

Some colleagues formed a whatsapp group a few days ago to help us more personally in case someone needs it. It is a complement to the [study] app. Anybody who wants to join will be welcome.

ID 192, man, 41 years

The connection between participants that was made in the group was so strong that the need arose to get to know each other outside of the study:

It would be good to meet someday, and not just those fromTarragona – everybody whowants to and can!

ID 548, woman, 54 years

In total, 10 people arranged a day to meet. As a separate whatsapp group was established, to which the research team did not have access, we do not know exactly how many people got together. We do know that it was satisfactory because they talked about organizing a second one for the people who could not attend:

A great get-together!!!! At the end of summer, another one, ehhhh?

ID 647, woman, 41 years

## Discussion

### Principal Findings

This study found that a chat integrated into a mobile app was a useful tool for offering social support and sharing emotions, information, or coping strategies to smokers in the process of quitting the habit. To our knowledge, this was the first qualitative descriptive analysis of a chat included in an app aimed at people in the *action* stage of change who were trying to quit smoking.

The analysis of the chat messages showed that it was an active forum used by participants to exchange information, concerns, and social support. Some of the emotions described by Eckman appeared in the chat [8]. In the first phases, users show sadness and fear a relapse. These withdrawal symptoms peak within the first weeks and last for about 2 to 4 weeks [17]. As they progressed in the process, participants moved to more positive emotions such as joy and even euphoria. In addition, in the more advanced phases the participants minimized the risk of relapse as considered themselves to be past that phase.

Values and beliefs are essential to initiate the smoking cessation process as well as to maintain abstinence. As in previous studies [18-21], we found that health and family, including concerns about a family member’s health or illness, or not willing to be a bad example for children, were primary reasons for quitting smoking. Moreover, the health benefits of smoking cessation are an important motivation to maintain abstinence as well as passing health checkups revisions, mainly to obtain a 0 in the co-oximetry.

Although cigarette taxes have shown dramatic increases in Spain, following the European Union legislation (Council Directive CD 2011/64/EU), participants infrequently reported money as a reason for quitting smoking. When they did report it, money appeared as a motivator in combination with other elements such as health. These results differ from a previous study in France in which money was the most reported reason for quitting smoking [20]; France suffered a dramatic increase of cigarette taxes between 2003 and 2004 [20]. The difference in effect between France and Spain in tax increases may be mediated through the height of the increase that in France was acute and large, not stepwise as Spain. However, our results concurred with a study performed in Spain in which money was not a main reason for quitting smoking [21]. Moreover, a 2006 study in Spain found that the introduction of a tax on manufactured cigarettes did not affect smoking prevalence in men and had a weak effect in women [22].

Most information provided in the chat was related to avoiding weight gain. Although weight varies greatly after quitting cigarettes, a published meta-analysis found that about 16% of quitters lost weight and 13% gained more than 10 kg [23]. The participants in our study believed it to be impossible to avoid weight gain. Smokers, and particularly women, have a high level of weight concerns that influence the likelihood of initiating a smoking cessation process [24]. However, the users of our chat believed that smoking is worse than increased weight.

Within the chat group, we observed that people who had a relapse were embarrassed and, although some sought the help of the group, some participants might not have asked for help because they felt guilty about deceiving themselves and above all for deceiving the group at the same time.

Within its social support, the group also offered emotional and informational support. We found similar results in other research, such as the study by Coulson et al, which indicates that group members offer informational and emotional support [25]; Ko et al [26] suggested that self-disclosure in blogs or Facebook is beneficial to users in obtaining social support and establishing or maintaining friendships [27]. However, in our study, various members of the group felt a need to meet each other and organized a time to get together. This could be because *the group* acquired such importance that its components wanted to connect in person.

### Clinical Implications

The Tobbstop app was designed to accompany the process of quitting for the first 90 days, the most critical days for a possible relapse. Participants who succeeded in abstaining from smoking used the chat to help newcomers providing advice, information, and emotional support. However, previous studies found that more than half of the messages from the support group were posted during the first months of the smoking cessation process indicating that people require more support in the first steps of quitting [3].

We found an important online social support community that complemented the information and support provided in primary health care consultations and other resources (expert patient of tobacco cessation, group activities, and community activities) in the first phases of smoking cessation programs. Moreover, online support groups have the potential to provide a unique opportunity for health professionals to learn about the experiences and views of individuals.

Online social support from an established group during the change process has several benefits. Participants are not restricted by the temporal, geographical, and spatial limitations typically associated with face-to-face groups; individuals can send and receive messages at any time of the day or night. In addition, online support groups may bring together a more varied range of individuals to offer diverse perspectives, experiences, opinions, and sources of information.

The emotional support obtained from the app may help some people deal with relapses. Little is known about how online discussions transform into real-life behavioral changes [28]. Efficacy is a concern because a recent review concluded that no robust evidence exists of the effectiveness of online peer-to-peer support groups [28]. An important next step is to assess the efficacy of online app forums by conducting randomized controlled trials.

New technologies and, more specifically, chat as a channel of communication may be able to help us to create groups of people who are engaged in the same process such as smoking cessation. The chat group can provide support and help 24 hours a day.

### Limitations and Strengths

We neither know the reasons why some participants did not use the chat nor what their comments might have been; it is possible that some users only read posts and did not contribute to them. For those users, it would be useful to determine which channel of communications would work best. A descriptive analysis has been done. It would be interesting to conduct a more in-depth and interpretative analysis according to sex, age group, studies, and other characteristics considered. According to the study protocol, participants who relapsed to tobacco consumption were removed from the Tobbstop app [12]. Those who relapsed were dismissed from the study and could not use the app, so we lack information to determine their emotions and feelings before the relapse, a process contemplated within Prochaska and DiClemente stages of change [14].

Among the strengths of the study was the interaction between participants who were in different phases of the process. Some people were just starting and others had already gone 180 days without smoking. A person who has already passed through a given stage will show empathy, respect, and confidence in others’ abilities and reinforce the social support. In addition, the chat showed a diverse and pluralistic discourse.

### Conclusions

The results of this study suggest that a chat integrated into a mobile app can be a useful tool for smokers who are in the process of quitting. In our study, the app offered social support and a space where participants shared concerns, information, and strategies This type of online social support could complement the information and support provided in primary health care consultations and other resources in smoking cessation programs.

## Abbreviations

IDIAP: Investigación en Atención Primaria
IQR: interquartile range
